# Sexual and reproductive health literacy of school adolescents in Lao PDR

**DOI:** 10.1371/journal.pone.0209675

**Published:** 2019-01-16

**Authors:** Viengnakhone Vongxay, Femke Albers, Souksamone Thongmixay, Maliphone Thongsombath, Jacqueline E. W. Broerse, Vanphanom Sychareun, Dirk Rombout Essink

**Affiliations:** 1 University of Health Sciences, Vientiane, Lao PDR; 2 Vrije Universiteit Amsterdam, De Boelelaan, the Netherlands; 3 Provincial Health Department, Huaphan Province, Lao PDR; Università degli Studi di Perugia, ITALY

## Abstract

**Rationale:**

Adolescent pregnancy in Lao PDR is the highest in Southeast Asia. It leads to negative health and social consequences in young people. It is anticipated that this problem is partly caused by limited sexual and reproductive health literacy (SRHL), leading to poor sexual and reproductive health (SRH) decisions. Based on the concept of health literacy, SRHL goes beyond knowledge and behavior and is the self-perceived ability of an individual to access the needed information, understand the information, appraise and apply the information into informed decision making for a good way to contribute to sexual and reproductive health. It is not only knowing (knowledge) and doing (behavior), but it is the process of individual’s thought on an SRH problem before taking an action. The aim of this study was to measure SRHL among school-going adolescents aged 15–19 and to determine factors associated with SRHL.

**Method:**

We conducted a cross-sectional study in rural and urban areas of Lao PDR in 2017. Respondents completed a self-administered questionnaire with five parts: socio-demographic, personal health, SRH knowledge and behavior, SRHL, and functional literacy. We calculated the SRHL score based on the HL-EU index and used descriptive statistics to determine the score and levels. Then we used bivariate statistics and multiple linear regression to identify factors associated with SRHL in these adolescents.

**Result:**

Among 461 respondents, 65.5% had inadequate SRHL. Scores were positively and significantly associated with several factors, including: school location (β: 3.218; p<0.001), knowledge on SRH and attending SR class in school (p:0.010—p<0.001), and functional literacy on condoms, which reflected how respondents understood the use of condoms (β: 0.871; p<0.001).

**Conclusion:**

Because most school adolescents had inadequate SRHL, comprehensive sexual education and enabling information as well as service access for adolescents are essential to ensure that adolescents can access, understand, appraise and apply good SRH knowledge in decision-making to benefit their own health.

## Introduction

Problems related to sexual and reproductive health among adolescents are a major concern in low-income countries. For example, teenage pregnancy (TP) and child marriage are more likely to occur in poor, low-educated and rural communities [1]. Worldwide, around 11% of all pregnancies are in adolescents aged 15–19 years and about 95% of these pregnancies occur in low- and lower middle-income countries [2]. TP is associated with adverse health outcomes in mothers and newborns and is the leading cause of death in girls. The younger the girl, the higher the chance of poor outcomes [3]. Moreover, adolescents are at greater risk for unsafe abortion, young maternal death, violence and Sexually Transmitted Infections (STIs) including HIV/AIDS, as well as the social consequences of school expulsion, child marriage and poverty. All of this maintains the vicious circle of living in poverty, low education and therefore having a higher risk of TP [3]. The capacity of adolescents to make informed decisions based on correct knowledge on sexual and reproductive health (SRH) is one of the factors that contributes to the prevention of SRH problems [4].

Sexual and reproductive health education is a protective factor for adolescent pregnancy, adverse SRH outcomes and negative social consequences [5–8]. However, knowledge is not sufficient; social skills and competences to promote and maintain healthy life are also important. These competences together are defined as Health Literacy [9], which is defined as having “the knowledge, motivation and competences to access, understand, appraise and apply health-related information within the healthcare, disease prevention and health promotion setting” [10]. In recent years, more research has focused on the importance of health literacy and the association of its lack with adverse health behavior and outcomes, especially in people with a low education and income [5, 10, 11]. However, most studies focused on word recognition and/or reading comprehension [12, 13] and general health literacy [14], rather than particularly on SRH literacy (SRHL). SRHL goes beyond knowledge and behavior and reflects how the motivation and competences to access, understand, appraise and apply sexual and reproductive health-related information to cope with SRH problems. Studies in the specific area of SRHL are required, as the quality and accessibility of SRH information directly affects the capacity of adolescents to access, understand, appraise and apply SRH information to maintain their SR health [15, 16].

This study focuses on Lao People’s Democratic Republic (Lao PDR). In Lao PDR teenage pregnancy is still a problem: 19% of women become mothers before the age of 18, which is the highest rate in South-east Asia. A relatively high proportion of 15.1%, of maternal deaths occur in young girls [17]. The low general health literacy [18] and the high TP rate in Lao PDR indicates that there is a lack of sexual knowledge and effective sexual education among adolescents. This can be partly attributed to low rates of school attendance, where adolescents usually get SRHR education [2, 17, 19]. The country is still struggling with the availability of youth friendly services thus adolescents have limited access to high quality health care services, which can impede their ability to put knowledge into practice [20, 21]. Good SRHL is therefore very important for Lao adolescents’ health and future, because it can lead to better decisions for their health [5, 22].

The aim of this study was to measure the SRHL in adolescents attending school in Lao PDR, as a start to gain insight into their current SRH literacy level and to find out how it is related to socio-demographic factors, sexual knowledge and behavior, health-lifestyle, and functional literacy. Knowledge gained from this study will contribute to the design of health interventions to improve the SRH literacy of teenagers and thus to reduce TP rates.

## Methods

### Study design

We conducted a quantitative cross-sectional study from January to June 2017, in three provinces (Huaphan, Vientiane. and Attapeu), representing the north, center and south of Lao PDR; using lottery sampling of one province for each part of the country. Then we selected one urban district and one rural district in each province using the same method.

### Study population

High school students, aged 15–19 years, both male and female were the study population. We sampled 461 school adolescents through multi-step sampling from among 2,400 identification numbers (IDs) of students from 12 upper secondary schools who did not have class at the time of data collection. The sampling of targets started from purposive sampling for public high schools in selected districts. After that, we recruited high school students into questionnaire sessions to form sampling lists; students were free to refuse if they did not want to participate regardless of the reason (2,400 students were invited; only 28 students refused to participate due to personal reasons, such as wanting to go home when no teaching class, having another activity, feeling tired or ill). Then we screened for eligibility by age range and completion of the SRHL questions, as this is the principle part of the questionnaire and key objective of the study, to finalize the sampling frame (1,841 students). Thereafter, we used the software SPSS to run the selection by IDs random for 461 representatives.

### Study tools

We used a self-administered structured questionnaire comprising five parts: (1) Socio-demographic, (2) Personal health-lifestyle, (3) SRH knowledge & behavior, (4) SRH literacy and (5) Functional literacy on condoms. We adopted the questions of part 1 and 2 from the Lao version of a health literacy study given to first year university students in Lao PDR in 2014, available at the University of Health Sciences, Lao PDR. The questions of part 3 were derived from the WHO illustrative questionnaire for interview-surveys with young people (available on website: http://www.who.int/reproductivehealth/topics/adolescence/questionnaire.pdf). The questions in part 4, we adapted based on 47 questions of the Lao version of HL study in first year university students of Lao PDR in 2014, adjusted to fit with the theme of SRHL in adolescents. Each question had a scoring scale from 0 to 4, which stands for ability to access, understand, appraise and apply SRH information from scale of ‘don’t know = 0’; ‘very difficult = 1 to very easy = 4’. Example questions: *“How easy is it for you to find information about contraceptive methods that teenagers can use*?, *… to understand the information about contraception*?, *… to judge how to avoid unintended pregnancy*? *… to decide if you need or do not need the contraception*?*”*. For the questions in part 5, we formulated them based on the Lao language functional information in leaflets and on packages of a well-known brand of condoms in Lao PDR, the ‘Number One’. The students were given a condom box to refer to when answering this question. We validated and piloted the questionnaire with 40 high school adolescents in urban and rural settings, not the study sites. On the basis of the pilot, the list of questions in the SRHL part was revised from the original 60 questions to only 45 questions, with Cronbach’s alpha = 0.9 (Pearson correlation). Before the students started answering the questionnaires in the classrooms, we asked them to sit far from each other to maintain confidentiality.

We calculated the SRHL score using the formula “Index-score = (mean—minimal value of mean) * (50/3)”, scaled by 4 levels: inadequate: 0–25, problematic: >25–33, sufficient: >33–42 and excellent: >42–50. Both the formula and the scales were adopted from the European health literacy survey (HLS-EU-Q47) method [22].

### Data analysis

We used descriptive statistics to describe the characteristics, score and level of SRHL. To identify factors possibly associated with SRHL score, we performed two-step inferential statistics; bivariate analysis and multiple linear regression, to determine the potential factors (p<0.05) with regression coefficient to predict the strength and direction of the association, and 95% confidence interval to assess the precision of the prediction. We included all independent variables with p < 0.100 in the final model.

### Ethical considerations

The Ethics Committee for Health Research of the University of Health Sciences, Lao PDR, reviewed and approved the research proposal before the data collection took place. During data collection, we introduced ourselves and explained to all students about consent and confidentiality, that participation was voluntary and anonymous, not interrupting learning hours, and they had the right to refuse to answer any questions without any consequences to them. We also gave every student a one-page information sheet, which described the purpose of study and contact information of the principle investigator, in case they had any question afterwards. As every public school provides knowledge about SRH through regular learning curriculum and youth activities and parent consent was not a concern in the context of settings, we asked for consent directly from each participant. However, we did an official request to the schools’ principals two weeks before conducting the data collection and all allowed us.

## Results

**Fig 1:**
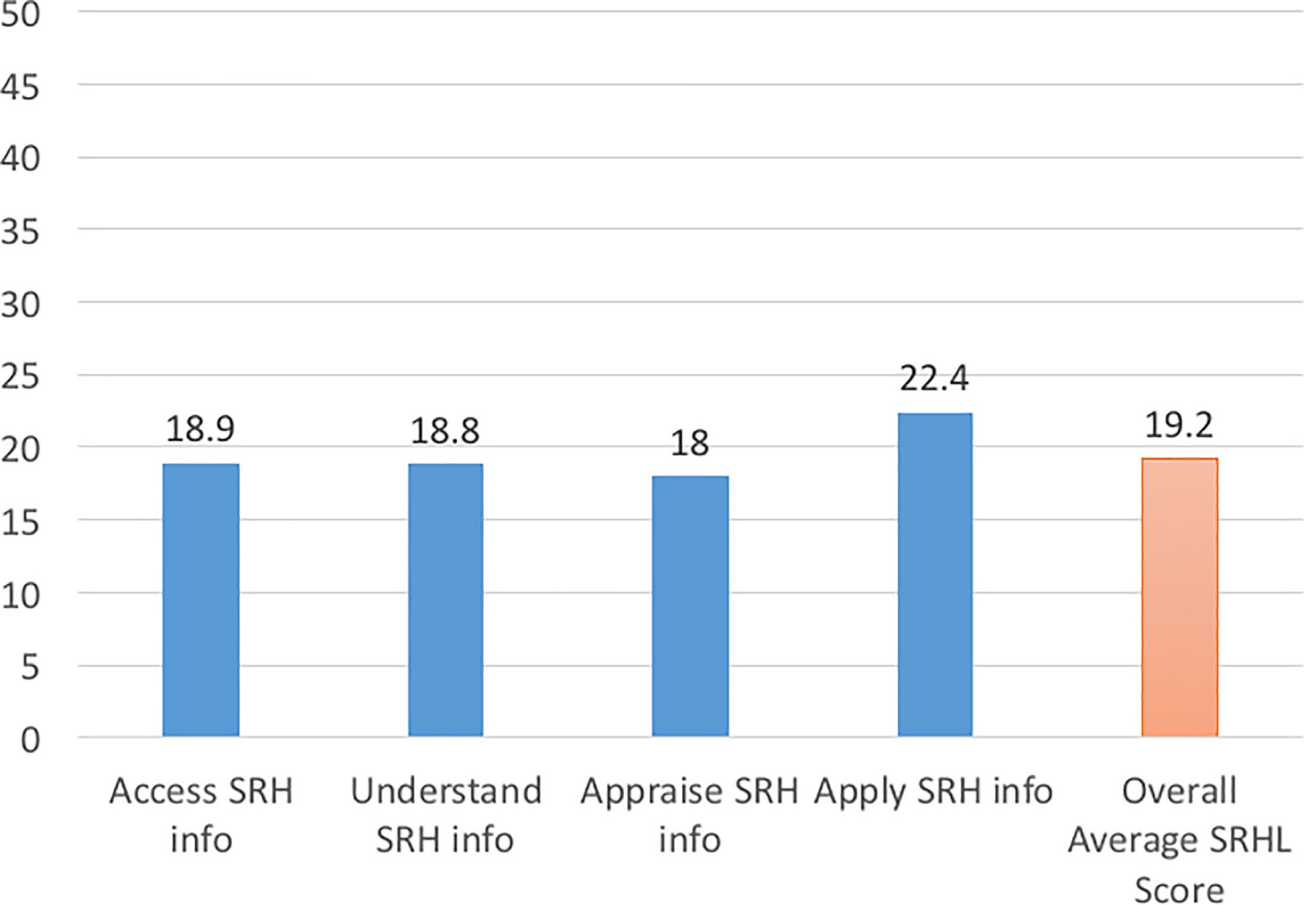
Mean score of SRHL in high school adolescents. Mean score of ability to access, understand, appraise and apply SRH information; index: 0–50.

Among the 461 respondents, 284 represented school adolescents in urban and 177 in rural areas (Table 1). Fig 1 shows that the overall mean SRHL score was 19.2/50. Most of the adolescents had scores in the range of ‘inadequate’ SRHL level (Table 2).

**Table 1:** Social demographic and personal health characteristics in relation to SRHL score.

Characteristics of adolescent (variables^(a)^)		Total students n = 461	SRHL score	Association with SRHL
		Freq (%)	Mean/Med.	p-value
**Socio-demographic**				
School location	Urban	284 (61.6)	21.0/22.9	**<0.001**^**(M)**^
	Rural	177 (38.4)	16.2/15.8	
Gender	Boy	217 (47.1)	20.0/20.5	0.167^(M)^
	Girl	244 (52.9)	18.5/19.8	
Spoken language in family	Lao	443 (96.1)	19.1/20.3	0.602^(M)^
	Ethnic	18 (3.9)	20.0/23.6	
Having part-time job	Yes	42 (9.1)	21.2/22.2	0.154^(M)^
	No	419 (90.9)	18.9/20.0	
Marital status	Single	436 (94.5)	19.1/20.1	0.735^(M)^
	In-union	25 (5.5)	19.5/24.7	
Living with parents	Yes	420 (91.1)	19.0/20.0	0.306^(M)^
	No	41 (8.9)	20.4/23.6	
Family member works related to health	Yes	69 (15.0)	21.4/23.6	**0.053**^**(M)**^
	No	392 (85.0)	18.8/19.7	
Having internet access	Yes	394 (85.9)	19.8/20.8	**0.001**^**(M)**^
	No	65 (14.1)	15.0/13.9	
Affordability to access general health care	Very difficult	29 (6.3)	16.0/15.8	0.130^(K)^
	Fairly difficult	89 (19.3)	18.4/19.4	
	Fairly easy	289 (62.7)	19.5/21.4	
	Very easy	54 (11.7)	20.6/20.3	
Age^(b)^: mean = 16.9 (±1.0)	min:15—max:19	-	-	**0.049**^**(S)**^
**Personal health**				
Self-estimate health status	Very bad	10 (2.2)	21.9/21.4	0.412^(K)^
	Bad	3 (0.7)	18.3/21.1	
	Fair	88 (19.1)	18.5/20.1	
	Good	283 (61.4)	19.8/20.8	
	Very good	77 (16.6)	17.5/16.9	
Having Disability	Yes	12 (2.6)	18.7/20.3	0.792^(M)^
	No	449 (97.4)	19.2/20.3	
Having chronic disease	Yes	30 (6.5)	18.5/21.2	0.772^(M)^
	No	431 (93.5)	19.2/20.3	
Feeling when compare health to people of same age	Worse than majority	22 (4.8)	19.3/19.4	0.119^(K)^
	Worse than a few	82 (17.8)	20.4/22.1	
	Average	198 (43.0)	18.0/18.9	
	Better than a few	128 (27.7)	19.4/21.1	
	Better than majority	31 (6.7)	22.7/26.7	
Had health problem in last 6 months	Yes	215 (46.6)	19.9/20.8	0.165^(M)^
	No	246 (53.4)	18.5/19.9	
Smoking status	Never Smoker	413 (89.6)	19.2/20.3	0.916^(K)^
	Ever smoker	27 (5.9)	18.9/19.7	
	Current Smoker	21 (4.5)	18.5/17.5	
Alcohol drinking in the past 12 months	Never drink	111 (24.1)	17.4/18.1	0.357^(K)^
	< once a month	234 (50.7)	19.9/21.4	
	Once a month	77 (16.7)	19.0/18.9	
	Once a week	36 (7.8)	19.7/20.8	
	> once a week	3 (0.7)	20.9/19.2	
Physical exercise > 30 minutes in last month	Not at all	53 (11.5)	16.9/16.1	0.155^(K)^
	1–2 times/month	81 (17.6)	19.1/18.6	
	1–2 times/week	158 (34.3)	18.7/20.0	
	Almost every day	169 (36.7)	20.4/22.2	

(a) Any variables not shown in Table 3 were not statistically associated with level of SRHL of adolescent in the univariate analysis (p>0.05)

(b) Mean age of school adolescent in urban area was 16.8 (±1.1); min: 15 –max:19 and in rural area was 17.2 (±0.9); min: 15 –max:19. Based on spearman’s correlation, age was found to be negatively correlated with SRHL score (correlation coefficient = -0.092; p:0.049).

(M) Mann-Whitney U Test

(K) Kruskal-Wallis Test

(S) Spearman’s correlation.

**Table 2:** Sexual and reproductive health literacy scores in relation to knowledge and behavior among school adolescents.

Characteristics of adolescent (variables^(a)^)		Total students n = 461	SRHL score	Association with SRHL
		Freq (%)	Mean/Med.	p-value
**SR knowledge and behaviors**				
Learning about Puberty from*	Teachers	278 (60.3)	18.9/19.7	0.371^(M)^
	Others	183 (39.7)	19.6/21.7	
Learning about SR system from*	Teachers	313 (67.9)	19.3/20.3	0.735^(M)^
	Others	148 (32.1)	18.9/19.7	
Learning about relationship-respect from*	Teachers	188 (40.8)	17.9/18.6	**0.020**^**(M)**^
	Others	273 (59.2)	20.0/21.9	
Attending SR subject class***	Regularly	331 (71.8)	20.0/21.4	**0.006**^**(M)**^
	Rarely/never	130 (28.2)	17.0/17.8	
Joining SR education or youth related activity ***	Regularly	100 (21.7)	20.3/22.6	0.208^(M)^
	Rarely/never	361 (78.3)	18.9/20.0	
SRH knowledge score**	0–4^(g)^	-	**-**	**<0.001**^**(S)**^
know that first sex can lead to pregnancy**	Know	104 (22.6)	22.7/24.0	**<0.001**^**(M)**^
	DK	357 (77.4)	18.2/19.2	
Know that first sex cannot stop body growing**	Know	98 (21.3)	24.7/25.3	**<0.001**^**(M)**^
	DK	363 (78.7)	17.7/18.0	
Know that masturbation does not bring serious health problem**	Know	82 (17.8)	23.7/25.1	**<0.001**^**(M)**^
	DK	379 (82.2)	18.2/19.2	
Sex on half-way between periods is most likely to get pregnant**	Know	118 (25.6)	22.9/24.6	**<0.001**^**(M)**^
	DK	343 (74.4)	17.9/18.6	
Had sexual intercourse before***	Yes^(c)^	64 (13.9)	19.9/22.3	0.508^(M)^
	No	397 (86.1)	19.1/20.0	
Ever been pregnant/made girl pregnant***	Yes	5 (1.1)	23.5/25.3	0.324^(M)^
	No	456 (98.9)	19.1/20.3	
Think that condom is the safest for sex****	Yes	198 (42.9)	21.5/22.8	**<0.001**^**(M)**^
	No	263 (57.1)	17.4/17.8	
Ever use health service related to SRH***	Yes	16 (3.5)	14.8/13.8	**0.075**^**(M)**^
	No	445 (96.5)	19.3/20.5	
Ever use online/hotline counseling related to SRH***	Yes	9 (2.0)	20.7/21.7	0.708^(M)^
	No	452 (98.0)	19.1/21.1	
Best contraceptive against pregnancy is…… method ****	Sterilization	71 (15.4)	23.0/24.7	**0.001**^**(M)**^
	Temporary	390 (84.6)	18.5/19.4	
**SRH literacy**				
Functional literacy on condoms^(e)^	mean score: 6.6(±2.5)	-	-	**<0.001**^**(S)**^
**Overall mean score level of SRHL**^**(f)**^	Excellent	5 (1.1)	-	-
	Sufficient	30 (6.5)	-	-
	Problematic	124 (26.9)	-	-
	Inadequate	302 (65.5)	-	-

*Sources of SRH knowledge

**Knowledge on SRH

***SRH related behaviors

****Perception on contraceptive

(a) Any variables not shown in Table 3 were not statistically associated with SRHL score of adolescent in the univariate analysis (p>0.05)

(c) Mean age of their first sex was 16.0 (±1.4) and the lowest age of first sex found in this study was 10 years old (min = 10—max = 19). 28.4% of them reported did not use any contraceptive method in their first sex, 64.9% used condoms, 6.7% used other methods (pills withdrawal and safe period).

(e) Functional literacy on condoms was scored based on 10 questions (with correct-incorrect answer) related to the use of condom indicated on condom pack-box. Mean score that the school adolescent got was 6.6(±2.5); min: 0 –max:10. (mean in urban: 6.7; mean in rural: 6.3). Based on spearman’s correlation, functional literacy score was found positively correlated with SRHL score (coefficient = 0.340; p <0.001).

(f) Mean score levels of SRHL are based on EU-HL index 0–50 (42<50: Excellent; 33<42: Sufficient; 25<33: Problematic; 00–25: Inadequate). In this study, the SRHL mean score is 19.2 and median is 20.3.

(g) The SRH know score ranged from 0–4, sum-up from 4 knowledge-questions (Incorrect = 0; correct = 1). Mean score = 0.9(±1), median = 1, min = 0 –max = 4. This means average SRH knowledge score of school adolescent was only 22.5%, which is quite low. Based on spearman’s correlation, SRH knowledge score was found positively correlated with SRHL score (coefficient = 0.330; p <0.001)

(M) Mann-Whitney U Test; (K) Kruskal-Wallis Test

(S) Spearman’s correlation

Table 1 shows a significantly higher mean and median SRHL score from schools in an urban setting and having Internet access. Adolescent age and having a family member who works in the health area were marginally associated with better SRHL (p: 0.049 & p: 0.053). Characteristics of personal health did not show any significant difference in SRHL.

Table 2 shows the results for SRHL among the school adolescents and related knowledge and behaviors. Most received SRH information from their teachers. It was noteworthy that 14% of them reported having experienced sexual intercourse. From 461 students, 2 girls had ever been pregnant and 3 boys had ever made girls pregnant. The average functional literacy score of school adolescents was 6.6/10. Groups having a higher SRH knowledge and higher functional literacy on condoms also had significantly higher SRHL scores. Specifically in the knowledge section, we found significantly higher SRHL scores from adolescents *who learn about gender relationship & respect from other sources than school teachers (family*, *friends*, *activities*, *internet*, *etc*.*)*, *who attend SR subject class regularly and who have a higher SRH knowledge score* (see Table 2).

Seven significant predictors were identified, which included *school site (urban vs*. *rural)* as demographic factor, *attending the sexual and reproductive subject in school class*, *knowledge about sex and body growing*, *knowledge about sex and menstruation cycle related to pregnancy*, *perceiving on condoms for safe sex*, *perceiving sterilization and other contraceptive methods against pregnancy as knowledge factors*, *and functional literacy on condoms* as *a skill factor*. So, demographical, knowledge and skill factors strongly contributed to the predictive model.

Table 3 shows the results of investigation into the factors associated with better or worse SHRL scores. Adolescents who attended schools in urban settings had significantly higher SRHL scores (β: 3.218; p<0.001). Respondents who had higher knowledge on SRH also had significantly higher SRH literacy scores (p: <0.001–0.010), such as adolescents who thought that ‘*condom is generally the safest for sex’* (β: 3.918), who thought that ‘*the best contraceptive method is sterilization’* (β: 4.550), who knew that ‘*sex half-way between periods is most likely to result in pregnancy* (β: 2.908)’, who knew that ‘*first sex cannot stop body growing’* (β: 4.549), and who ‘*attend SR subject class regularly’* (β: 2.434). Also, adolescents who had higher functional literacy about condoms had a significantly higher SRHL score (β: 0.871; p<0.001).

**Table 3:** Factors associated with level of SRHL among adolescents attending school.

Variables		Regression coefficient (β)	P-value	95%CI for (β)	
				lower	Upper
Constant		7.388	0.311	-6.924	21.700
**Socio-demographic**					
Adolescent in urban/rural school:	Urban	3.218	**<0.001**	1.456	4.981
	Rural	Ref.			
Having internet access:	Yes	1.429	0.269	-1.108	3.965
	No	Ref.			
Family member works related to health:	Yes	1.958	0.099	-0.372	4.287
	No	Ref.			
Age:	(15–19 yrs old)	-0.384	0.328	-1.154	0.387
**SRH Knowledge & Behaviors**					
Learning about relationship-respect from:	School teachers	-1.520	0.079	-3.217	0.176
	Others	Ref.			
Attending SR subject class	Regularly	2.434	**0.010**	0.595	4.273
	Rarely/never	Ref.			
SRH knowledge score*:	0–4	-	-	-	-
know that first sex can lead to pregnancy:	Know	1.034	0.322	-1.014	3.082
	DK	Ref.			
Know that first sex cannot stop body growing:	Know	4.549	**<0.001**	2.468	6.630
	DK	Ref.			
Know that masturbation does not bring serious health problem:	Know	2.175	0.056	-0.057	4.408
	DK	Ref.			
Sex on half-way between periods is most likely to get pregnant:	Know	2.908	**0.003**	0.991	4.825
	DK	Ref.			
Think that condom is the safest for sex:	Yes	3.918	**<0.001**	2.086	5.750
	No	Ref.			
Ever use health service related to SRH:	Yes	-1.566	0.494	-6.061	2.929
	No	Ref.			
Best contraceptive against pregnancy is:	Sterilization	4.550	**<0.001**	2.006	7.093
	Temporary method	Ref.			
**Functional Literacy**					
Functional literacy score on condoms	0–10	0.871	**<0.001**	0.519	1.222

Adjusted R^2^ = 0.291

* ‘SRH knowledge score 0–4’ was excluded from the model due to the collinearity statistics tolerance (0.000)

## Discussion

This study explored the sexual and reproductive knowledge and sexual behavior of adolescents in Lao PDR and measures their SRH literacy levels and factors affecting it. Data was collected using a new questionnaire that was developed, focusing on SRH, so that SRH literacy levels could be measured. The results reveal that most adolescents had inadequate SRHL. As this was the first time SRHL was measured, comparable literature is lacking. However, by proxy, the findings of general health literacy measurement in first-year university students in Vientiane also showed that the health literacy score was problematic, although with a higher mean score (26.38) than we found [18]. Previous studies found associations between age and HL while we did not, possibly because of a smaller age-range in our adolescent sample [23, 24].

When looking at predictive factors for SRHL levels, we found that attending schools in urban settings had a significant positive influence on the SRHL. This finding was consistent with previous literature [23, 25]. SRH knowledge and skill (functional) factors were also strongly and positively associated with higher SRHL scores, suggesting that individual knowledge and competency influences the SRHL level; the finding is consistent with previous reports in the literature [9, 10, 26, 27].

Although we did not find studies on SRHL in similar low-income countries, the findings might be predicted from known data on the poor reproductive health outcomes in large number of adolescents in Lao PDR [5]. Lower levels of SRH literacy were found among adolescents with less actual knowledge on sexuality and reproduction, who also had lower scores on the functional health literacy test.

Previous research among boys in school, between 16 and 19 years in Lao PDR found a two times higher prevalence of experience with sexual intercourse, while contraceptive use seems consistent between the studies [28]. The mean age at first sexual intercourse was lower than found in previous studies [17, 29], while research in the north of Lao PDR found a younger mean age and higher prevalence of sexual intercourse [30]. As this study focuses on adolescents attending school, differences in the sample population, such as age, ethnicity and educational level, are probably the most relevant factors causing these differences. The focus in this study and a previous one on school boys might have resulted in the finding of lower prevalence of sexual activity, because more of the study population would be of lower ages [28]. The result also shows that peer education has a positive influence on SRHL in adolescents, more than learning only from school teachers. However, the main source of SRH knowledge in our study population was the school teachers, in contrast to a previous study in Lao PDR, in which the major information sources were television and radio [28]. Our findings also show a very small number of pregnancy-experienced adolescents; this is likely owed to school regulations that require expulsion of pregnant adolescents. This is also worthy to consider for a further study specifically to this group, as there might be more pregnancy-experienced adolescents who have already been expelled from school.

There was an unanticipated finding in the SRHL section of the study, in that many adolescents responded ‘don’t know’ to many questions. In contrast to the original questionnaire used in the previous study, our questionnaire was self-administered. We therefore needed a ‘don’t know’ option. However, the literature also provided critical reasons why respondents answer ‘don’t know’: (1) respondents actually do not have the relevant knowledge, (2) respondents believe their answer does not meet the needs of the question, and (3) respondents choose not to provide the answer for personal or social reasons [31]. Considering that not knowing is not the only reason for a student to choose that option, it would be good to conduct a specific study on comprehensive SRHL tool validation and testing in a specified context; with more consideration on the length of the questionnaire, the layout of the questions and answer options [32, 33].

In this study, we noted a few limitations that might be useful to consider for further research. Demographically, the sample might not exactly represent the national demographics, such as the number of male students, which were slightly underrepresented in our study [17]. Also, we did not recruit adolescents already out of school who might have very different knowledge and experiences. The results should however be generalizable to adolescents attending upper secondary school in Lao PDR. Culturally, as having premarital sex is prohibited by school regulations, and could result in being expelled, respondents might have given socially desirable answers to the questions related to sexual experience. Discussing about sex is also a cause for shyness in this age group. We did try to account for these concerns by giving enough physical space between participants to ensure privacy and requesting silence, and explaining that there are no right or wrong answers, and no consequences to them. They might also not be fully reassured that the school would not see and act on their answers. Thus, we cannot rule out that despite these measures students still felt insufficiently ‘safe’ to answer these questions truthfully. That may account for the low number reporting having had sexual experience. In analysis, although we included many relevant characteristics that might influence SHRL, there might be other factors that we did not measure but may contribute to the SRHL; e.g. household income or parental educational level. We excluded these because we expected that most respondents would not know the exact information, leading to unreliable results. The final predictive model reported here explained only 29.1% of the variance in SRHL score. In future studies, these additional characteristics should be explored.

The results reported here add to our understanding of the knowledge and capacity of school-going adolescents in Lao PDR regarding sexual and reproductive health. Our findings emphasize the importance of good quality, curriculum-based sexual education programs in secondary schools. Policymakers should focus on training teachers and developing sustainable sexual education programs in schools, which can be incorporated in the curriculum [34]. The focus of these programs should be more on competencies such as good health literacy. Currently the curriculum is limited to the biology classes on the human reproductive system, and taught in 6^th^ grade (17–18 yrs). Previous interventions have proven that such inputs can have a positive impact on behaviors and significantly delay the initiation of having sex as well as increasing contraceptive use [35, 36].

There is no one best intervention for every country; appropriate interventions should be based on the local context and specific target group [33, 37, 38]. Evidence to support development of good health strategy and action to create an enabling environment for adolescents to access SRH information and services is therefore essential [39, 40]. Our findings emphasize the need for SRH service improvement and regular SRHL monitoring in adolescents.

Future research might focus on a more extensive qualitative investigation on SRHL and especially could include youth who are no longer in school, to get more insight into their specific problems and SRHL. That information is essential to guide potential health interventions to reduce the TP rate, and to improve the sexual knowledge and skills of all teenagers in Lao PDR for their own health and protection.

## Implications and contribution

This study offers insight into the inadequate status of SRH knowledge, behavior and literacy among Lao adolescents. Within study sites, extensive and diverse topics in the questionnaire as well as data collection across geographically and demographically distinct areas in Lao PDR provide a broad basis for further research and for policy development and revision.

## Supporting information

S1 File Data set_ASRHL_Anonymous. (SAV)
